# Knowledge, attitude and practice of emergency contraceptive among women who seek abortion care at Jimma University specialized hospital, southwest Ethiopia

**DOI:** 10.1186/1472-6874-12-3

**Published:** 2012-03-12

**Authors:** Tatek Tesfaye, Tizta Tilahun, Eshetu Girma

**Affiliations:** 1 Kotebe Hospital, Addis Ababa, Ethiopia; 2 Department of Population and Family Health, Jimma University, P.O. Box 5093, Jimma, Ethiopia; 3 Department of Health Education and Behavioral Sciences, P.O. Box 5093, Jimma, Ethiopia

## Abstract

**Background:**

In Ethiopia maternal mortality rate is very high more than one in five women die from pregnancy or pregnancy related causes. The use of contraceptives to prevent unwanted pregnancies and unsafe abortion is an important strategy to minimize maternal mortality rate. Among various forms of contraception, emergency contraceptives are the only one that can be used after sexual intercourse offering chance to prevent unwanted pregnancy. The aim of this study was to assess the knowledge, attitude and practice of emergency contraceptive among women who seek abortion care at Jimma University specialized hospital (JUSH).

**Methods:**

Institution base cross-sectional study on knowledge, attitude and practice of emergency contraceptive was conducted at JUSH from April to June, 2011Data was collected using structured questionnaire and analyzed using SPSS version 17.0.

**Results:**

In this study 89 women were interviewed. More than half of them (48) were from urban area and 41 were from rural area.46 (51.7%) of them were single. Of all the respondents only nine women had awareness about emergency contraceptive. Seven of the women mentioned pills as emergency contraception and only two of them mentioned both pills and injectable as emergency contraception. All of them have positive attitude towards emergency contraception but none of them have ever used emergency contraceptives.

**Conclusion and recommendation:**

The finding revealed pregnancy among women of 15-19 years was very common. The knowledge and practice of emergency contraception is very low. But there is high positive attitude towards emergency contraceptives. Since there is much deficit on knowledge of women on emergency contraceptives, in addition to making them accessible; programs targeted at promotion and education of emergency contraceptives is helpful to prevent unwanted pregnancy.

## Background

History of emergency contraceptive dates back to the 1960's when physicians in the Netherlands administered estrogen extracts to 13 years old girl who had been raped in mid cycle [1]. Emergency contraceptive can prevent pregnancy when taken shortly after unprotected sex. Currently there are four food and drug administration of America approved products on market. Three of these products are approved for prevention of pregnancy when taken within 72 hrs after unprotected sex. Adults may purchase all of these methods without prescription and individuals who are at least 17 years old may purchase one of these methods Plan B (one step- without a prescription). The fourth product, Ella can be taken up to 5 days after unprotected sex; it is available by prescription [2].

Levenorgestrel only pill and combined oral contraceptives are the most common emergency contraceptives available in Ethiopia [3]. It has been known since the mid 1970's that high doses of oral contraceptives given postcoital are effective in preventing pregnancy. The uzepe method, the typical regimen, involves taking one dose containing estrogen and progestin (100 microgram of ethinyl estradiol and 1 mg of Levenorgestrel or 0.5 mg of levonovgestrel) with in 72 hrs after unprotected intercourse, followed by another dose 12 hrs later. The most recent data confirm that compliance with this regimen reduces the risk of pregnancy by 75% [4].

Unintended pregnancy poses a major challenge to reproductive health of young adults in developing countries. Some young women who had unintended pregnancies obtain abortion. Many of which are performed in unsafe condition and others carry their pregnancies to term, incurring the risk of morbidity and mortality higher than those for adult women [5].

Given increasing adolescent sexual activity and decreasing age at first sex in developing countries the use of contraceptives to prevent unwanted pregnancies and unsafe abortion is especially important [6]. It might take some time for Ethiopian abortion law to take another courageous step towards legalizing abortion on social and economic grounds. Meanwhile, however, the issuance of the Technical and Procedural Guidelines for Safe Abortion by the Ministry of Health, at the heel of the new law is indeed encouraging. To address the large number of maternal deaths caused by unsafely-performed abortions, the Ethiopian Parliament liberalized the indications for legal abortion in 2004. Indications for legal abortion include: cases of rape or incest, if the woman has physical or mental disabilities, to preserve the woman's life or her physical health, or if the woman is a minor who is physically or mentally unprepared for childbirth. No consent from a spouse, partner or parent is required to obtain a legal abortion and no requirements exist for legal reporting or documenting rape or incest as a prerequisite for obtaining a legal abortion. Thus, this law opens some room to have safe abortion care at the health facilities [7,8].

Like other governmental institutions in Ethiopia Jimma University Specialized Hospital render medical abortion treatment as per abortion law of the country women. According to DHS 2005 Ethiopia, 16% young women had sex by age 15 and 35% women had sex by age 18 years. Amongst women aged 15-19 years, 17% become mothers or are currently pregnant with their first child. Maternal mortality in this age group when expressed in proportion to female deaths it accounts for 12.1% [9].

Among various forms of contraception emergency contraceptive are the only one that can be used after sexual intercourse offering chance to prevent unwanted pregnancy. Therefore the objective of this study was to determine knowledge, attitude and practice of emergency contraception among women who seek abortion at Jimma University Specialized Hospital, Southwest Ethiopia.

## Methods

### Study area and period

This study was conducted at Jimma University Specialized Hospital (JUSH) found in Jimma town, south western Ethiopia. It provides inpatient service in 6 different specialties. There are other three specialty clinics which provide outpatient service only. Gynecology and obstetrics department is one of the major departments that provide both inpatients and outpatient services having two wards [obstetrics and gynecology wards, which accommodate 80 beds. Out of which 12 beds are for abortion cases. From November 2010 to November 2011 1137 patients admitted as gynecological case in gynecology ward [10].

### Study design

A cross -sectional study was conducted on women who seek abortion care at JUSH.

### Source population

All women admitted to gynecology ward with a diagnosis of abortion and seeking medical abortion.

### Study population

All women admitted to gynecology ward with a diagnosis of abortion and seeking medical abortion within the study period.

### Measurements

We have adopted the instrument from a previous study on emergency contraceptive. The instrument had internal consistency for knowledge questions with cronbach's alpha = 0.86 and the reliability for attitude questions was cronbach's alpha = 0.72 [11].

After pretest was done among ten women from other health facility, face to face interview using a structured questionnaire was employed for study. The inquiry includes variables which included the following categories. Independent variables were age, religion, educational level, marital status, occupation, source of information while the dependent variables were Knowledge of emergency contraceptive of the women, attitude towards emergency contraceptive, and practice on emergency contraceptive use.

### Inclusion and exclusion Criteria

All women in the study population except women who could not able to respond to the questionnaire because of severely ill (could not hear and speak).

### Data collection

Face to face interview through structured questionnaire was done. The questionnaire was kept in the ward and patients were interviewed on arrival. Before the data collection medical interns who work in gynecology ward were trained, in order to have common understanding on each question. The data collection was supervised by the principal investigator.

### Data analysis and presentation

The Data was analyzed by SPSS for windows version 17.0. Descriptive statistics chi-square tests were done and significance of tests was decided at p-value 0.05. Tables and graphs were used to depict results.

### Operational definitions

Knowledge- Awareness about the types, time limit to be taken after unprotected sex and dosage of emergency contraceptives. Attitude - The way to which clients are thinking or behaving towards emergency contraceptive. Practice - Trend of use of emergency contraceptives among women who seek abortion care.

### Ethical consideration

Ethical clearance was obtained from Jimma University College of Public Health and Medical Sciences. Ethical issue was considered in all steps of the research. Detailed explanation about the objective, purpose and benefit of the study was given to the study population. Study participants full cooperation and verbal consent was taken.

## Results

### Socio-demographic characteristics

**Figure 1:**
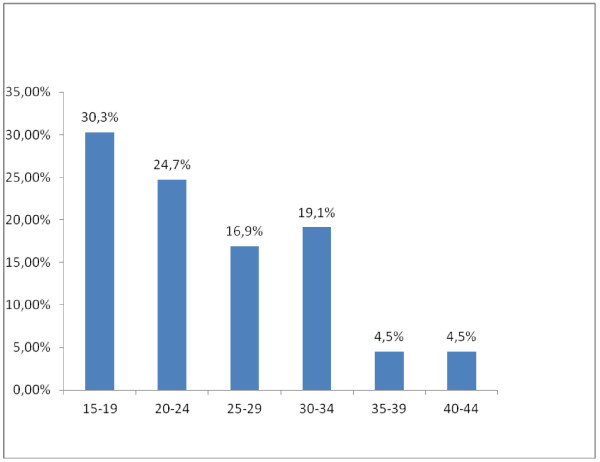
**Age Distribution of the Women Who Came For Abortion Care at JUSH Gynecology Ward May-June 2011**.

A total of 89 women were interviewed during the study period. Of all respondents 27(30.3%) were in the range of 15-19 years of age (Figure 1). 63(70.3%) were Oromo in ethnicity. Majority 65(73%) were Muslim in religion. More than half 49 (55.1%) were from urban area. Majority 46(51.7%) were single and 46(51.7%) had no child. 22(24.7%), 39(43.8%) were illiterate and elementary class (grade 1-8) complete respectively in their education (Table 1). Only 10(11.2%) were government employee. Regarding to their residence, 49.1% were from rural and the rest were from urban places.

**Table 1:** Socio- Demographic Characteristics of Women Who Came For Abortion at JUSH Jimma, Southwest, Ethiopia 2011

	Number	Percent
**Religion**		

Orthodox	17	19.1

Protestant	7	7.9

Muslim	65	73.0

**Marital status**		

Single	46	51.7

Married	39	43.8

Divorced and widowed	4	4.5

**Number of living children**		

One	12	13.5

Two	16	18.0

Three and above	15	16.8

None	46	51.7

**Occupation**		

Housewife	35	39.3

Student	23	25.8

Government employee	10	11.2

Housemaid	21	23.6

**Educational status**		

Cannot read and write	22	24.7

1-8	39	43.8

9-12	18	20.2

12+	10	11.2

N = 89

### Reproductive health background characteristics

Out of 89 women 48(53.9%) had their first sex at the age of less than 18. 44(49.5%) of them become pregnant at the age of less than 18 for the first time (Table 2). Majority 65(73%) of the women never used contraceptives of any kind. Among those who used contraceptive, 10(41.7%) used injectable contraceptive. Only six women had previous history of induced abortion while of them nine had spontaneous abortion (Table 3). For the current pregnancy 50(56.2%) and 39(43.8%) were induced and spontaneous type of abortion.

**Table 2:** Age at First Sex and Contraceptive Use among Women Who Seek Abortion at JUSH Southwest, Ethiopia 2011

Characteristics	Number	Percent
**Age at 1^st ^sex[n = 89]**		

< 15	18	20.2

15-18	30	33.7

18+	41	46.1

**Ever used contraceptives [n = 89]**		

Yes	24	27

No	65	73

**Type of contraceptives used [n = 24]**		

OCP	6	25

Inject able	10	41.7

Implant	2	8.33

More than one	6	25

**Total**	**24**	**100**

**Table 3:** Past History of Pregnancy and Related Characteristics among Women Who Seek Abortion at JUSH Jimma, Southwest, Ethiopia 2011

Characteristic	Number	Percent
**Induced abortion [n = 89]**		

No	83	93.3

Yes	6	6.7

**No of abortions [n = 6]**		

One	5	77.8

Two	1	22.2

**Place of abortion [n = 6]**		

Self infliction	1	22.2

Health institutions	4	57.1

Traditional healers	1	20.7

**Spontaneous abortion**		

No	80	89.9

Yes	9	10.1

**No of abortions [n = 9]**		

One	7	71.4

Two	2	28.6

### Knowledge, attitude and practice on emergency contraceptives

#### Knowledge on emergency contraceptives

80(89.9%) women had no information about emergency contraceptive. Of them nine women know emergency contraceptive. Seven and two of the nine mentioned progesterone only pills, both progesterone only pills and injectable types of emergency contraceptive respectively. Two of them heard from their friends, two from radio and five of women point out more than one source of information. Seven women identified the correct time limit to take emergency contraception. Only two of them know the doses to be taken and nobody knows the time interval between the doses. None of them know the time limit for IUCD to be inserted. 4 (44.4%), 2 (22.2%) and 3 (33.3%) stated pharmacies, nongovernmental health institutions and both respectively (Table 4).

**Table 4:** Knowledge of Emergency Contraceptive by Different Socio Demographic Groups at Southwest, Ethiopia 2011

	Yes Number	No Number	***P -value ***df = 1
**Religion [n = 89]**			

Orthodox	9	8	0.001
	
Protestant	0	8	
	
Muslim	0	64	

**Place of origin [n = 89]**			

Urban	9	40	0.004
	
Rural	0	40	

**Occupation [n = 89]**			

Housewife	0	35	0.015
	
Student	1	22	
	
Government employee	6	4	
	
Housemaid	2	19	

**Ever used contraceptive [n = 89]**			

Yes	5	19	0.04
	
No	4	61	

**Educational level [n = 89]**			

**Illiterate**	0	22	0.001
	
**1-8**	0	39	
	
**9-12**	3	15	
	
**12+**	6	4	

#### Attitude and practice on emergency contraceptives

Nine of the women had positive attitude towards emergency contraception, but none of them used emergency contraception. Occupational status, previous history of contraceptive use had significant statistical association with knowledge on emergency contraceptive but the others do not show an association [p = 0.05].

## Discussion

Unintended pregnancy poses a major challenge to reproductive health of youth in developing countries. Some young women who had unintended pregnancies obtain abortion. Many of which are performed in unsafe conditions and others carry their pregnancies to term, incurring the risk of morbidity and mortality higher than those for adult women [12]. Emergency contraceptive can prevent pregnancy when taken shortly after unprotected sex. The finding shows that 48(48.5%) women become pregnant for the first time at the age less than 18 years. This is comparable to the result obtained at Harar town about 41% of 675 women had their first pregnancy between the age of 15-19 years. Use of modern contraceptive methods is low when compared to the result obtained in Harar town which is 24(27%) in this study and 295 (37.5%) from Harar [13].

Of the respondents 18 (20.2%), 30 (33.7%) and 41 (46.1%) were less than 15 years, 15-18 years and greater than 18 years of age at first sex respectively. This is similar with the national figure which is 16% young women had sex by age 15 and 35% women had sex by age 18 years [9]. For the current pregnancy 50 (56.2%) and 39 (43.8%) were induced and spontaneous type of abortion. Among them 51 (57.3%) women came in need of medical abortion. Of them 40 (78.4%) reported that they become pregnant after being raped. This result is much or similar with study from selected health facilities in Ethiopia where three-fourth of patients had spontaneous abortion and one fourth (25.6%) of them had induced abortion [14]. This is much greater when contrast to result obtained from Survey of unsafe abortion on 1075 women in selected health facilities in Ethiopia where rape account only for 3% of all pregnancies that ended in abortion [14]. The possible reason for such great difference could be sample taken in this study is small in number and from only one health facility.

Nine women have heard about emergency contraceptive, seven of them describe 72 hours as the time limit to take emergency contraceptive. When asked about specific types emergency contraceptives. Seven of them responded pills only and two of them both pills and injectable whereas only two women answered correctly dosing and time interval between the doses (which is 2 and 12 hours respectively). These study findings were comparable study from Adigrat in Ethiopia from the total 902 patients, only, 11 patients had the knowledge of emergency contraception (1.2%) in addition, and only 3 patients were given emergency contraceptive after forced sexual intercourse (2.4%) [15]. None of them used emergency contraceptive. This is unlike with a study done on 774 students at Addis Ababa University, about 43.5% [95% CI, 40.0-47.0%] of the students said that they have heard about emergency contraception, 279 (82.8%) depict pills and 115 (34.1%) intrauterine contraceptives. About 53% [95% CI 4.19% [95%CI 3.1 -6.4%] respondents reported that they had used emergency contraceptive methods previously [12]. And still the result is much less when compared to study done in Nigeria among female undergraduates where 58% of respondents had heard of a product that could be used to prevent pregnancy after unprotected sex. Of the 510 woman who were aware of emergency contraception only 18% correctly identified 72 hrs at the limit time for the method's use [6]. This difference could be attributed to higher proportion of rural population 41 (46.1%), less literacy level 22 (24.7%) and less number of study population. Place of origin, literacy level, and religion, shows a significant association between knowledge of emergency contraceptive. However, occupational status, previous history of contraceptive use have slight association [p = 0.05].

### Limitation of the study

The study suffers from the usual limitation of a cross sectional study. The study also did not discover any information from the service providing side of emergency contraceptive in the health facilities. Moreover, the nature of the sensitivity of the issue (abortion) and past history on abortion might affected by recall bias. But, we had made an effort get possible information and made the interview with respondents private.

## Conclusion

The study disclosed that there are a high number of females under the age of 18 years who practiced sexual intercourse which may result unintended pregnancy. Moreover, this unwanted pregnancy urges women to practice unsafe abortion which in turn leads to maternal death. The study also revealed that there is a low trend of use of modern contraceptives. As a whole it can be concluded that Knowledge, and practice of emergency contraceptive is low whereas all had positive attitude towards emergency contraceptive use. In the face of high number of induced abortion and young age pregnancy awareness of emergency contraceptive is very low. Much more effort should be done on information education and communication of awareness and practice of modern contraceptives. Particularly focus should be given on emergency contraception to females so that able to decrease unintended pregnancy and abortion cases. In addition, works should be done at the level of health posts and health centers to enhance awareness and practice cost and consequences of abortion on women's` health. Furthermore studies exploring the positive attitude-practice gap should be done on women of abortion and rape prevalence to see the cause and effect relationship clearly.

## Competing interests

The authors declare that they have no competing interests.

## Authors' Contributions

TT and TT designed the study, analyzed the data and drafted the manuscript. EG drafted and critically reviewed the manuscript. All authors read and approve the final manuscript.

## Pre-publication history

The pre-publication history for this paper can be accessed here:

http://www.biomedcentral.com/1472-6874/12/3/prepub
